# A Budget Impact Model for Paclitaxel-eluting Stent in Femoropopliteal Disease in France

**DOI:** 10.1007/s00270-012-0494-x

**Published:** 2012-10-17

**Authors:** Erwin De Cock, Marc Sapoval, Pierre Julia, Greg de Lissovoy, Sandra Lopes

**Affiliations:** 1 Peri- and Post-Approval Services, United BioSource Corporation, Carrer Torrent del Remei, 5-11, 4°-2a, 08023 Barcelona, Spain; 2 Department of Cardiovascular and Interventional Radiology, Hôpital Européen Georges Pompidou, Université René Descartes, 20 rue Leblanc, 75015 Paris, France; 3 Cardiovascular Surgery Department, Hôpital Européen Georges Pompidou, Université René Descartes, 20 Rue Leblanc, 75015 Paris, France; 4 Department of Health Policy and Management, Johns Hopkins Bloomberg School of Public Health, Hampton House 325, 624 North Broadway, Baltimore, MD 21205 USA; 5 Health Economics and Reimbursement, Cook Medical, Sandet 6, 4632 Bjaeverskov, Denmark

**Keywords:** Budget impact model, Drug-eluting stent, Paclitaxel-eluting stent, Peripheral artery disease, Superficial femoral artery

## Abstract

**Purpose:**

The Zilver PTX drug-eluting stent (Cook Ireland Ltd., Limerick, Ireland) represents an advance in endovascular treatments for atherosclerotic superficial femoral artery (SFA) disease. Clinical data demonstrate improved clinical outcomes compared to bare-metal stents (BMS). This analysis assessed the likely impact on the French public health care budget of introducing reimbursement for the Zilver PTX stent.

**Methods:**

A model was developed in Microsoft Excel to estimate the impact of a progressive transition from BMS to Zilver PTX over a 5-year horizon. The number of patients undergoing SFA stenting was estimated on the basis of hospital episode data. The analysis from the payer perspective used French reimbursement tariffs. Target lesion revascularization (TLR) after primary stent placement was the primary outcome. TLR rates were based on 2-year data from the Zilver PTX single-arm study (6 and 9 %) and BMS rates reported in the literature (average 16 and 22 %) and extrapolated to 5 years. Net budget impact was expressed as the difference in total costs (primary stenting and reinterventions) for a scenario where BMS is progressively replaced by Zilver PTX compared to a scenario of BMS only.

**Results:**

The model estimated a net cumulative 5-year budget reduction of €6,807,202 for a projected population of 82,316 patients (21,361 receiving Zilver PTX). Base case results were confirmed in sensitivity analyses.

**Conclusion:**

Adoption of Zilver PTX could lead to important savings for the French public health care payer. Despite higher initial reimbursement for the Zilver PTX stent, fewer expected SFA reinterventions after the primary stenting procedure result in net savings.

## Introduction

Peripheral artery disease (PAD) is a progressive, lifestyle-limiting condition with prevalence in the range of 3–10 %, although it increases to 15–20 % in patients over 70 years of age [1]. Risk factors such as smoking, obesity, diabetes, hypertension, and age suggest that the prevalence of PAD in modern societies could be increasing [2]. Although most patients with PAD do not have symptoms, a proportion will develop symptomatic disease that manifests as pain in the leg muscles with exercise (intermittent claudication) and pain at rest, or skin lesions such as ulcers and gangrene (critical limb ischemia) [1].

Management of PAD is conditioned on a number of factors such as patient symptoms, patient characteristics (such as gender, age, smoking, obesity, cardiovascular risk factors), response to lifestyle and risk factor modification, and long-term prognosis [3–6]. Bypass surgery has been widely used to treat patients with critical limb ischemia and at a risk of limb loss, or those with longer or more complicated lesions. However, as a major surgical procedure, it has been associated with high morbidity and mortality as well as considerable resource use [7, 8]. Endovascular therapies are now commonly used to achieve revascularization in patients with intermittent claudication or critical limb ischemia where conservative therapy has not been successful. Within the endovascular treatment spectrum, percutaneous transluminal balloon angioplasty (PTA) is the simplest and least resource intensive procedure. PTA is, however, associated with high 1-year restenosis rates that worsen with longer and more complex lesions. Reported results vary widely, with 1-year patency rates for PTA ranging from 37 to 80 % [1, 9]. More recently, self-expanding nitinol bare-metal stents (BMS) have demonstrated improved patency results, yet restenosis remains a limitation [9–12].

Success in coronary artery intervention has led to investigation of drug-eluting stents in the superficial femoral artery (SFA). The Zilver PTX drug-eluting stent (Cook Ireland Ltd., Limerick, Ireland) is a nitinol stent with a polymer-free paclitaxel coating designed to treat the above-the-knee femoropopliteal arteries. Zilver PTX was recently evaluated in a large multicenter, multinational randomized, controlled trial and a complementary multinational, multicenter, single-arm clinical study [13–15]. The technology met the safety (12-month event-free survival) and effectiveness (primary patency) end points in these studies. Furthermore, results from the Zilver PTX randomized study demonstrated superior performance compared to PTA and compared to the identical bare-metal Zilver stent [13].

At a time when health care costs continue to escalate and budgets are under scrutiny, it is important to understand the impact of new technologies on health care spending. Whereas some innovations will increase the overall cost of treating patients, while improving survival and/or quality of life, the a priori perception that all innovation adds cost to the system is often the result of a short-term assessment of the impact on health care budgets, rather than evaluating the medium- to long-term cost consequences. The present budget impact assessment was conducted to inform the reimbursement decision for Zilver PTX in France. It demonstrates the 5-year budget impact of a progressive shift from BMS to Zilver PTX.

## Materials and Methods

The budget impact model was constructed in Microsoft Excel. Because the model was originally developed as part of a reimbursement application to the French national health care authorities, the perspective is the third-party payer. The impact on the public health care budget of gradually adopting Zilver PTX in France was compared to the scenario where only BMS would be available in situations where stent placement would be the standard of care, with the model only considering BMS as a class as opposed to specific BMS types. The published decision on Zilver PTX reimbursement in France, as with many other implant devices listed in the French Liste des Produits et Prestations Remboursables (LPPR), is valid for a 5-year period [16]. Therefore, a 5-year model time horizon was chosen, with a new patient cohort entering the model each year from 2012 until 2016. Net budget impact was expressed as the difference in cost between the scenarios where Zilver PTX is progressively adopted versus BMS only.

### Eligible Population

French national hospital episode statistics from the Agence Technique de l’Information sur l’Hospitalisation (ATIH) were used to estimate the patient population [17]. As a first step, the total number of lower limb peripheral stenting procedures performed in 2010 was determined [17].

**Fig. 1:**
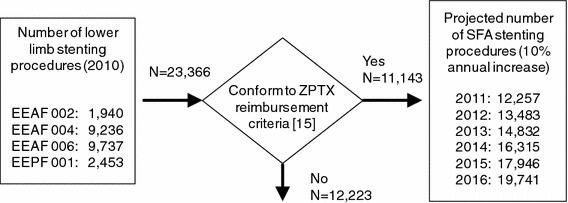
Build-up of model patient population for SFA stenting procedures. Classification Commune des Actes Médicaux (CCAM) codes for lower limb stenting procedures [26]: *EEAF 002* Percutaneous transluminal dilatation of an artery of the lower limb with transluminal dilatation of the common iliac artery and/or of the ipsilateral external iliac artery with stent implantation, *EEAF 004* Percutaneous transluminal dilatation of an artery of the lower limb with stent implantation, *EEAF 006* Percutaneous transluminal dilatation of several arteries of the lower limb with stent implantation *EEPF 001* Percutaneous transluminal recanalization of an artery of the lower limb with stent implantation

However, the published reimbursement decision states that Zilver PTX reimbursement should be limited to patients with symptomatic atherosclerotic occlusive disease of the lower extremities, when used to treat lesions (length ≤14 cm) in the femoropopliteal arteries above the knee having a reference vessel diameter ranging between 4 and 9 mm, after failure of PTA [18]. Furthermore, the identified relevant procedure codes describe lower limb stenting procedures in general, but are not specific to any one artery in particular. According to the French authorities, the target patient population for SFA stenting with Zilver PTX was estimated to be about half (48 %) of the total number of lower limb stenting procedures performed as per 2008 data [18]. This percentage was applied to the total number of relevant procedures performed in the latest year for which ATIH data are available (2010). It was assumed that the target population would grow at a flat 10 % annual rate in subsequent years, which was informed by the percentage annual growth in procedures up to 2010. According to these estimations, the total patient population eligible for SFA stenting in 2012 is 13,483 patients. The build-up of the model patient population is summarized in Fig. 1. In 2012, the impact of the reimbursement decision is expected to result in a market penetration rate of 15 %, or 2,022 patients. Moreover, it is expected that the annual growth rate in adoption of the new technology over the time horizon of the budget impact model is constant and equals 5 % per year after 2012, to yield a cumulative market penetration of 35 % by 2016.

### Model Structure

A new cohort of patients eligible for initial stenting enters the model each year, and patients in that cohort are treated either with Zilver PTX or BMS. As a result of restenosis of the treated lesion or lesions, a patient may need a reintervention at some point in time. The model used target lesion revascularization (TLR) rates for Zilver PTX and BMS in order to estimate the number of reinterventions and associated costs each year. Therefore, for any given year, the total number of patients needing a reintervention includes patients who have entered the model in that year as well as patients who received the initial stenting procedure in a previous year. The model assumes that a patient is not eligible for more than one reintervention procedure and does not die over the 5-year model time horizon. In the event of needing a reintervention, patients are treated with one of the following procedures: PTA; stenting or bypass surgery. The distribution of reintervention options was estimated on the basis of the observed distribution of reinterventions from the Zilver PTX single-arm clinical study, which enrolled 787 patients internationally (Cook Medical, data on file). The total costs of treating the target population with Zilver PTX or BMS over 5 years were compared to the costs of treating the same patient population if only BMS were available, in order to assess the overall budget impact of introducing Zilver PTX in the French public health care system.

### Clinical Data

In the absence of a head-to-head comparison of Zilver PTX and BMS at the time of model development, the 12- and 24-month TLR rates were extracted from the published literature on BMS [10, 11, 19] and from 2010 interim data from the Zilver PTX single-arm clinical study [14]. Interim data from the single-arm study were used because this was the trial with the longest-term (24-month) data for TLR and were the only published data available. Published data on 12-month TLR from the randomized study comparing safety and effectiveness of Zilver PTX with PTA and provisional BMS placement in SFA patients [13] were used in a scenario analysis.

The performance of the Zilver PTX stent was compared with BMS in the treatment of femoropopliteal lesions on the basis of published BMS data through subset analyses of the Zilver PTX single-arm study population data with matching inclusion/exclusion criteria for each individual BMS study (Table 1) [14].

**Table 1:** Literature comparison for TLR rates

Study	Inclusion criteria	BMS	Zilver PTX^a^
DURABILITY [10]	No in-stent restenosis	21 % at 12 month (*n* = 134)	6 % at 12 month (*n* = 474)
	Lesion length ≤14 cm		
	Rutherford 2–4		
FAST [11]	De novo lesions: length 1–10 cm	15 % at 12 month (*n* = 127)	6 % at 12 month (*n* = 282)
	Multiple lesions <10 cm total		
	≥70 % stenosis diameter		
RESILIENT [19, 20]	No in-stent restenosis	13 % at 12 month (*n* = 153)	6 % at 12 month (*n* = 467)
	Lesion length <15 cm		
	Rutherford 1–3		
		20 % at 24 month (*n* = 153)	9 % at 24 month (*n* = 235)

*TLR* target lesion revascularization, *BMS* bare-metal stent, *Zilver PTX* Zilver PTX drug-eluting stent (Cook Ireland Ltd., Limerick, Ireland)

^a^TLR rates for Zilver PTX were calculated from matching registry subset analyses. The inclusion criteria within each of the BMS published studies were matched for the Zilver PTX single-arm study [14]

All three BMS studies reported 12-month TLR rates [10, 11, 19]. Additionally, the RESILIENT study reported 24-month TLR data [20]. The TLR rates for Zilver PTX in each of the matching registry subpopulations, based on interim analysis, were 6 % at 12 months [14]. For BMS, an average 12-month TLR rate of 16.3 % was calculated across all three studies to serve as the base case value for BMS as a class (Table 2). At 24 months, the interim TLR rate for Zilver PTX was 9 % for the subset of patients matched with the RESILIENT study (Table 1) [14]. For BMS, an average 24-month TLR rate of 21.9 % was calculated on the basis of the difference observed between 12- and 24-month TLR rates in the RESILIENT and Zilver PTX studies (Table 2). More recent 12-month data for the three matching registry subpopulations indicated lower TLR rates (4–5 %) for Zilver PTX; thus, the rates used in the model analysis are somewhat higher and therefore conservatively overestimate the need for reinterventions. The impact on model results of a reduction in the average TLR rates for BMS was explored in a scenario analysis using data for LifeStent from the RESILIENT study, which at the time of model development was the only study that reported 12- and 24-month TLR rates [2, 11, 20].

**Table 2:** TLR and distribution of reintervention options after Zilver PTX or BMS

Characteristic	Variable	Zilver PTX (%)	BMS (%)	Source
Cumulative TLR rate	Year 1	6.0	16.3	Zilver PTX: single-arm study data [14, 15]; BMS: calculated values
	Year 2	9.0	21.9	
	Year 3	15.0	27.9	Table 1 and expert opinion
	Year 4	21.0	33.9	
	Year 5	27.0	39.9	
Type of reintervention	PTA	56		Zilver PTX: Cook, Zilver PTX single-arm study, data on file; BMS: assumption
	Stent	32		
	Surgical	12		
	*If stent required*			
	Zilver PTX	25		Expert opinion
	BMS	75		
	*If surgical intervention*			
	Synthetic graft	28		Bradbury et al. [21]
	Patient’s vein	72		
	*If synthetic graft used, proportion by type:*			
	Linear or nonlinear textile implant <30 cm	60		Expert opinion
	Linear or nonlinear textile implant ≥30 and <70 cm	40		

*TLR* target lesion revascularization, *Zilver PTX* Zilver PTX drug-eluting stent (Cook Ireland Ltd., Limerick, Ireland), *BMS* bare-metal stent, *PTA* percutaneous transluminal balloon angioplasty

No publications were found reporting the 3-, 4- and 5-year TLR rates after stent implantation. Therefore, those rates were extrapolated on the basis of clinical expert opinion that 5 years after initial stent placement, about 40 % of patients treated with BMS would have needed a reintervention. Absolute annual 6 % increments were applied to BMS from year 2 onward and the same 6 % increment after 24 months was also conservatively applied to the Zilver PTX group because no other data were available (Table 2). Thus, the clinical benefit of Zilver PTX is due to the lower TLR rates seen in the first 2 years after the primary procedure.

### Treatment Costs

To reflect the third-party payer’s perspective, only direct medical costs, covering inpatient treatment, were calculated. This was based on the reimbursement tariffs for both private and public hospitals, according to the French Groupe Homogène des Malades (GHM, a national diagnosis-related group system) [22] (Table 3). Because various GHM tariffs could apply to the episode of care for a patient requiring lower limb stenting, procedure codes relevant to lower limb stenting were identified, numbers of procedures were mapped to specific GHM codes for 2010 [17], and a weighted GHM tariff was calculated, taking into consideration the proportion of procedures conducted in public versus private hospitals. Reimbursement fees for consultants and anesthetists in private hospitals [26] were also taken into account and added to the respective private GHM tariff [22]. Costs were based on 2011 reimbursement tariffs, with variability in key cost inputs explored in sensitivity analyses. Weighted procedure tariffs of PTA and bypass surgery were calculated by the same methodology. Key model cost inputs are summarized in Table 3.

**Table 3:** Model cost inputs

Treatment	Cost (€)	Source
Zilver PTX	1,000.00	LPPR reimbursement tariff, code 3141310 [23]
BMS	841.52	LPPR reimbursement tariff; code 3183194 [24]
Linear/nonlinear textile graft <30 cm	336.61	LPPR reimbursement tariff; code 3171860 [25]
Linear/nonlinear textile graft ≥30 to <70 cm	541.50	LPPR reimbursement tariff; code 3189423 [25]
Linear/nonlinear textile graft ≥70 cm	658.58	LPPR reimbursement tariff; code 3122608 [25]
Stent implantation	3,082.00	Weighted cost, 2011^a^
PTA	3,225.00	Weighted cost, 2011^b^
Surgery	7,414.00	Weighted cost, 2011^c^

*Zilver PTX* Zilver PTX drug-eluting stent (Cook Ireland Ltd., Limerick, Ireland), *LPPR* Liste des Produits et Prestations Remboursables, *BMS* bare-metal stent, *PTA* percutaneous transluminal balloon angioplasty, *GHM* Groupe Homogène des Malades, *MI* myocardial infarction

^a^Relevant GHM codes are 05K061: vascular stent no MI, level 1; 05K062: vascular stent no MI, level 2; 05K063: vascular stent no MI, level 3; 05K064: vascular stent no MI, level 4; 05K06T: vascular stent no MI, very short stay 0 or 1 day [22]

^b^Relevant GHM codes are 05K131: Endovascular procedures without stent, age above 17 years, level 1; 05K132: Endovascular procedures without stent, age above 17 years, level 2; 05K133: Endovascular procedures without stent, age above 17 years, level 3; 05K134: Endovascular procedures without stent, age above 17 years, level 4; 05K13 J: Endovascular procedures without stent, age above 17 years, ambulatory [22]

^c^Relevant GHM codes are 05C101: Major vascular surgery, level 1; 05C102: Major vascular surgery, level 2; 05C103: Major vascular surgery, level 3; 05C104: Major vascular surgery, level 4 [22]

As previously mentioned, the expected direct cost of a reintervention was calculated on the basis of the observed distribution of reinterventions from the Zilver PTX single-arm clinical study (Table 2) (Cook Medical, data on file). The choice of reintervention option is assumed independent from the type of stent used for the primary procedure. Therefore, the expected cost of a reintervention after Zilver PTX and BMS primary stenting procedures was assumed the same.

### Analyses

In line with principles of good practice for budget impact analysis, no discounting was applied as the analysis presents financial streams over time [27]. The impact of relaxing key assumptions on model results was assessed through a number of scenario analyses, as summarized in Table 4.

**Table 4:** Scenario analyses

Variable	Scenario
Higher TLR rates after Zilver PTX stenting	12-month TLR 9.5 % [13]
	24-month TLR 12.5 % (estimated)
Lower TLR rates after BMS stenting	12-month TLR 12.7 % [12, 19]
	24-month TLR 20.0 % [20]
Zilver PTX market share at year 5	2012: 10 % to 2016: 30 %
30 % reduction in costs for primary stenting procedure and reintervention	Primary stenting: €2,158; reintervention: €2,774
30 % increase in costs for primary stenting procedure and reintervention	Primary stenting: €4,007; reintervention: €5,151
Increased number of Zilver PTX stents for primary stenting procedure	1.25^a^
Zilver PTX market share at year 5	2012: 20 % to 2016: 40.53 %^b^
8 % reduction in Zilver PTX and BMS tariffs	Zilver PTX: €920^c^; BMS: €774.20 (estimated)

*TLR* target lesion revascularization, *Zilver PTX* Zilver PTX drug-eluting stent (Cook Ireland Ltd., Limerick, Ireland), *BMS* bare-metal stent

^a^This assumes that a maximum of 25 % of cases would require two stents within this population, where the maximum lesion length is 14 cm

^b^To reach target population of 8,000 in 2016, in accordance with target population for Zilver PTX as defined by French authorities [18]

^c^In accordance with fixed tariff from March 30, 2013, onward as defined by French authorities [23]

## Results

Under the base case assumptions, the progressive adoption of Zilver PTX is cost saving from year 1 onward. The model estimated a cumulative 5-year budget reduction of €6,807,202 for a projected population of 82,316 patients, of which 21,361 received the Zilver PTX stent (Table 5).

**Table 5:** Total and net 5-year budget impact

Time	BMS only		Progressive adoption of Zilver PTX			Net annual budget impact (€)
	No. of procedures with BMS	Total treatment costs (€)	No. of procedures with Zilver PTX (% of total)	No. of procedures with BMS	Total treatment costs (€)	
Year 1 (2012)	13,483	61,632,893	2,022 (15 %)	11,461	61,125,303	−507,590 (−0.8 %)
Year 2 (2013)	14,832	70,748,351	2,966 (20 %)	11,865	69,801,480	−946,871 (−1.3 %)
Year 3 (2014)	16,315	81,028,788	4,079 (25 %)	12,236	79,708,287	−1,320,502 (−1.6 %)
Year 4 (2015)	17,946	92,337,269	5,384 (30 %)	12,562	90,577,880	−1,759,389 (−1.9 %)
Year 5 (2016)	19,741	104,776,598	6,909 (35 %)	12,832	102,503,749	−2,272,850 (−2.2 %)
Total over 5 years	82,316	410,523,900	21,361 (26 %)	60,956	403,716,697	−6,807,202 (−1.7 %)

Values are undiscounted; minus sign signifies reduction in annual budget

*Zilver PTX* Zilver PTX drug-eluting stent (Cook Ireland Ltd., Limerick, Ireland), *BMS* bare-metal stent

**Fig. 2:**
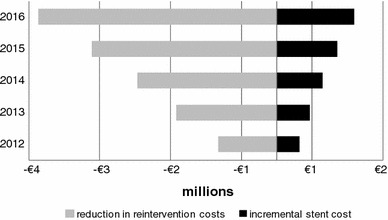
Net budget impact by year with Zilver PTX scenario versus BMS only. A *minus sign* indicates budget reduction; a *plus sign* indicates budget increase

The difference in stent cost between Zilver PTX and BMS is more than offset by the savings accruing from lower reintervention rates, and therefore less rehospitalization, in each of the 5 years after the primary procedure (Fig. 2). The model calculated that 2,572 reintervention events would be avoided over 5 years with the progressive adoption of Zilver PTX (or a reduction from 22,088 to 19,516 events). For a hypothetical patient starting treatment in 2012, estimated total cost, including primary stenting and expected reinterventions up to 5 years, was €5,503 when a BMS was used, compared to €5,152 with Zilver PTX (difference of €351).

**Fig. 3:**
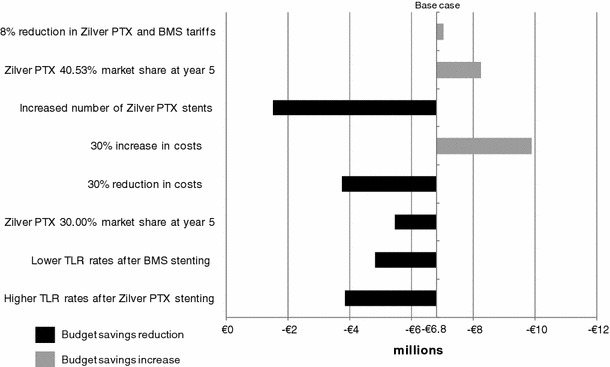
Five-year net budget impact with Zilver PTX scenario versus BMS only compared to base case. Scenario descriptions correspond with those in Table 4

Scenario analyses indicated that the adoption of Zilver PTX results in cumulative 5-year budget reduction regardless of scenario (Fig. 3). Increasing the average number of Zilver PTX stents to 1.25 per patient for primary stenting yields the lowest net budget reduction. Although the use of lower TLR rates for BMS or higher 12-month TLR rates for Zilver PTX decreases budget savings in each year, 5-year cumulative budget offsets remain substantial. Faster uptake of Zilver PTX further increases annual budget savings because of the expected increased reduction in reinterventions. Similarly, the higher the primary stenting and expected reintervention cost, the higher are the budget savings associated with the use of Zilver PTX. To illustrate, increased costs could result from a larger share of bypass surgery reinterventions, or the need for some patients to undergo two angioplasty procedures for in-stent restenosis, leading to higher hospitalization costs.

## Discussion

In September 2011, the French government published its decision to reimburse Zilver PTX [23] at €1,000 per stent, a 19 % premium over the BMS reimbursement level. A higher tariff could suggest that this new technology would result in increased health care spending. However, as this budget impact analysis demonstrates, the expected reductions in restenosis and TLR rates not only provide clinical benefit to patients, but also help to avoid costly rehospitalization. The model suggests that budget offsets start within the first year of adoption and are sustained over the 5-year model time horizon.

Zilver PTX has demonstrated reductions in restenosis and TLR rates compared to the more traditional treatments of PTA and/or BMS [10, 11, 13–15, 19, 20]. This is an important clinical breakthrough in the treatment of atherosclerotic SFA disease. In today’s health care environment of ever-increasing demands on limited financial resources, health care decision makers—physicians, payers, and patients—increasingly want to understand the economic ramifications of new technologies. More and more, the questions being asked are “is it worth it?” and if so, “at what cost?.”

There have been some attempts to assess the costs of treating patients with PAD. Mahoney et al. [2] compared the 2-year rates of vascular-related hospitalizations and associated costs in US patients with established PAD across patient subgroups. They concluded that the economic burden of PAD is high as a result of recurring hospitalizations and repeat revascularization procedures. A health care cost utilization study in the United States [28] attempted to assess the cost per day of patency after vascular reconstruction, and to determine if the initial cost savings of endovascular procedures were sustained over time. Although it concluded that the initial cost savings of endovascular therapies were not sustained, this analysis was limited to a 12-month time horizon and bundled all endovascular modalities (angioplasty, stenting, atherectomy or cryoplasty) into one group. The study was also limited by its retrospective nature and moderate sample size. In 2010, the authors of a review article on new SFA treatments [29] presented a hypothetical cost-calculation comparing the costs of using non-drug-eluting stents with drug-eluting stents. Although the review acknowledged the promise shown by preliminary clinical data for Zilver PTX, the cost analysis suggested that the stent would have an additional economic burden at 12 months after the procedure. However, the review has a number of limitations: (1) the underlying assumptions and methodology are unclear; (2) the sources for the hypothetical stent costs as well as for the reintervention profiles considered are not disclosed; and (3) the review used patency rates to calculate the number of repeat revascularization procedures, despite TLR rates being the more appropriate outcome.

As with any model, varying degrees of uncertainty exist around key model inputs. Model parameters were derived from previously reported studies and clinical trial data, supplemented by expert clinical opinion where published evidence was not available. Some of these uncertainties were: (1) the lack of randomized controlled trials directly comparing TLR rates of the Zilver PTX stent and current generation BMS; (2) the absence of long-term comparative results; (3) the assumption that the reintervention distribution was constant over time; (4) assumptions surrounding the number of stents used per SFA stenting procedure and the rate of uptake of Zilver PTX in France; and (5) the future level of GHM and LPPR tariffs. However, the uncertainties around the above parameters were addressed by conducting scenario analyses, the results of which confirmed the robustness of the model base case.

Like any other modelling study, which aims to reflect a complex real-world situation, this model has limitations. Firstly, the model population is restricted to the subgroup of lower limb atherosclerosis patients and shorter lesions (length ≤14 cm) and a reference vessel diameter between 4 and 9 mm, as per the French authority decision [18]. This may not allow extrapolation to other populations. Indeed, the analysis does not assess budget impact for patient populations with longer, more difficult lesions nor for Zilver PTX as a first-line treatment for diabetic patients and in-stent restenosis. Because it is particularly challenging to treat these subgroups of patients [30–33], it would be interesting to study the budget impact of the use of Zilver PTX in a wider population when more clinical evidence for such patients becomes available. Furthermore, the model only considers the patient population that is eligible for stenting and does not include populations where other treatments such as balloon angioplasty or bypass surgery are the standard of care. Although the analysis performed is specific to the impact of switching from BMS to Zilver PTX when treating femoropopliteal disease, there are also other new treatments that may be interesting to consider from a budget impact perspective, such as drug-eluting balloons [34].

Secondly, the model base case does not use randomized controlled trial data to compare TLR rates between groups. Although such data offer a higher level of evidence than single-arm study data, these were not available at the time of model development when only 12-month TLR rates were published for the primary stenting arm in the Zilver PTX randomized study (TLR = 9.5 %) [13]. This was used in a scenario analysis, and results were not much different compared to the base case. A 24-month update on the Zilver PTX randomized study reported 12- and 24-month TLR rates for the secondary randomization of provisional Zilver PTX versus provisional BMS, both after failed PTA (12-month TLR of 5.3 vs. 17.6 % and 24-month TLR of 10.8 vs. 23.1 %, respectively) [35]. The difference in 24-month TLR rates for provisional Zilver PTX and provisional BMS reported for the randomized study is very similar to the difference in the TLR rates from the interim analysis used in the budget impact model, which further strengthens the validity of the results.

Thirdly, in the absence of any long-term evidence (beyond 24 months), TLR rates for years 3, 4, and 5 were extrapolated assuming a constant 6 % absolute increase, which was informed by expert opinion estimating a cumulative 40 % TLR rate after primary stenting with BMS after 5 years. The validity of this assumption is confirmed by the recently published 3-year follow-up results from the RESILIENT trial, which indicated freedom from TLR of 75.5 % in the BMS group (equivalent to 24.5 % TLR compared to base case model average 3-year TLR of 27.9 %) [36].

Lastly, the model does not take into account mortality, as there was no clinical evidence suggesting a difference in survival between Zilver PTX and BMS. This has negligible impact on the reported budget reduction because the difference in cost between Zilver PTX and BMS groups is expected to remain constant with equal mortality.

Results from this study lend support to the argument that adoption of a somewhat more costly new technology can improve clinical outcomes while reducing expenditure, making both clinical and economic sense for the health care system to adopt the technology. To realize these benefits, it requires payers’ willingness to take a longer-term view of spending. It also demands that researchers and manufacturers involved with developing technology appreciate that health care budgets are not unlimited. These challenges can be met by both payers and manufacturers, as seen here with the adoption of the Zilver PTX stent in France, resulting in a reimbursement decision that can minimize financial hurdles for providers adopting the technology, ultimately benefiting the French patient.
